# Probiotics in the Intensive Care Unit

**DOI:** 10.3390/antibiotics11020217

**Published:** 2022-02-08

**Authors:** Alex R. Schuurman, Robert F. J. Kullberg, Willem Joost Wiersinga

**Affiliations:** 1 Center for Experimental and Molecular Medicine (CEMM), Amsterdam University Medical Centers, University of Amsterdam, 1105 AZ Amsterdam, The Netherlands; a.r.schuurman@amsterdamumc.nl (A.R.S.); r.f.j.kullberg@amsterdamumc.nl (R.F.J.K.); 2 Division of Infectious Diseases, Department of Medicine, Amsterdam University Medical Centers, University of Amsterdam, 1105 AZ Amsterdam, The Netherlands

**Keywords:** microbiome, probiotics, intensive care unit, dysbiosis, ventilator-associated pneumonia

## Abstract

The understanding of the gut microbiome in health and disease has shown tremendous progress in the last decade. Shaped and balanced throughout life, the gut microbiome is intricately related to the local and systemic immune system and a multitude of mechanisms through which the gut microbiome contributes to the host’s defense against pathogens have been revealed. Similarly, a plethora of negative consequences, such as superinfections and an increased rate of hospital re-admissions, have been identified when the gut microbiome is disturbed by disease or by the iatrogenic effects of antibiotic treatment and other interventions. In this review, we describe the role that probiotics may play in the intensive care unit (ICU). We discuss what is known about the gut microbiome of the critically ill, and the concept of probiotic intervention to positively modulate the gut microbiome. We summarize the evidence derived from randomized clinical trials in this context, with a focus on the prevention of ventilator-associated pneumonia. Finally, we consider what lessons we can learn in terms of the current challenges, efficacy and safety of probiotics in the ICU and what we may expect from the future. Throughout the review, we highlight studies that have provided conceptual advances to the field or have revealed a specific mechanism; this narrative review is not intended as a comprehensive summary of the literature.

## 1. Introduction

The gut microbiome harbors complex communities of bacteria which together fulfill a wide range of functions within the human body. A balanced gut microbiome enhances the host defense against infection by finetuning the local and systemic immune system [1,2], repressing enteric pathogens [3,4], and supporting epithelial barrier integrity [5]. Conversely, perturbation of the microbiome (called ‘dysbiosis’) appears to have detrimental effects on the host and is associated with a wide range of diseases [6]. This is of particular relevance in the intensive care unit (ICU) where patients with life-threatening conditions (such as respiratory failure, sepsis, myocardial infarction, cardiovascular procedures, intracranial hemorrhage and cerebral infarction) are treated [7]. 

The microbiome of such critically ill patients is continually assaulted by the disease itself and by iatrogenic effects of clinical intervention [2,8]. As a result, the gut microbiome of virtually all patients admitted to the ICU is severely disrupted [8,9,10]. These disruptions have associated with a multitude of negative consequences such as ventilator-associated pneumonia (VAP) and increased re-infection and re-admission rates [10,11,12]. The field of probiotics—the administration of selected, live bacteria that are of potential benefit to the host (see Section 1.1)—strives to address dysbiosis-related problems by reinforcing or reconstituting the gut microbiome, both in preventative and therapeutic approaches. In this review, we will first explore the causes and putative consequences of dysbiosis in ICU patients. Next, we summarize the experimental data, mainly comprising studies in mice, that support the rationale for probiotic administration in the critically ill. We proceed by discussing the current clinical evidence for probiotic intervention in the ICU, with a focus on the prevention of VAP in adults. Herein, we combine meta-analyses and a recent landmark clinical trial to evaluate the efficacy and safety of probiotics in the ICU. We close with a reflection on current opportunities and pitfalls in the field, and an outlook on the potential future positioning of probiotics in the ICU. 

### 1.1. A Brief Overview of Modalities Used in the ICU to Modulate the Gut Microbiome

Several (partly experimental) strategies are used in the ICU to modulate the microbiome in order to prevent or treat infections. Examples are the use of pre/synbiotics, probiotics, fecal microbiota transplantation (FMT) and antibiotic prophylaxis. Briefly, probiotics are selected ”live microorganisms that, when administered in adequate amounts, confer a health benefit on the host” [13]. Prebiotics are nutrients—often oligosaccharides—that can selectively feed certain bacterial colonies, while a combination of probiotics and prebiotics is called synbiotics. FMT comprises the transfer of a stool sample, autologous or from a donor, to a recipient in order to (re)introduce healthy bacterial flora. Probiotics can be administered in various ways, such as in a soluble powder or in pill form, which can contain billions of bacteria per dose. Often, such probiotics include bacterial strains from the *Lactobacillus* and *Bifidobacterium* species—sometimes genetically modified to have less virulent factors.

## 2. Gut Microbiota in Critically Ill Patients

### 2.1. Causes of Gut Microbiota Disruptions

Critically ill patients have a severely disturbed microbiome, characterized by a loss of diversity, depletion of commensal bacteria (e.g., *Ruminococcus*, *Pseudobutyrivibrio*, *Blautia*, *Faecalibacterium*) and domination by pathogens (e.g., *Enterococcus*, *Staphylococcus*, *Enterobacteriaceae*) [2,8,9]. These disruptions extend to kingdoms beyond bacteria (e.g., bacteriophages, eukaryotic viruses, fungi and protozoa) and enable the overgrowth of viruses and opportunistic yeasts [14]. A multitude of endogenous and iatrogenic factors contribute to these extensive alterations in the microbiota composition of ICU patients, including gastrointestinal dysmotility, shifts in intraluminal pH values, increased production of catecholamines, treatment with antibiotics, proton pump inhibitors, opioids and (par)enteral feeding [2,8]. In addition, infection of the gastrointestinal tract by pathogenic bacteria or viruses could drive microbiome alterations. Recently, several studies showed that SARS-CoV-2 can infect human enterocytes and that gut microbiota are disrupted during COVID-19 [15,16].

The exact effect of any of these disruptive factors on the composition of the gut microbiota varies highly per individual [17]. For example, Rashidi et al. analyzed 260 stool samples of patients with acute leukemia receiving multiple antibiotics and demonstrated that pre-treatment microbiota composition (specifically the earlier described health-promoting bacteria such as *Roseburia*, *Blautia* and *Eggerthella*) was the most important determinant of antibiotic-induced microbiota alterations. Even under intense antibiotic pressure, gut microbiota maintained a highly personalized composition [18]. Besides the iatrogenic changes and disruptive effects of critical illness itself, demographic variables also influence the microbiome during critical illness. In a cohort of 155 critically ill patients in the ICU, age and sex were associated with the differential abundance of a large number of bacterial taxa, while less associations were found between bacterial taxa and the length of ICU stay or disease severity (quantified by SOFA score) [19]. This is in line with a large study that analyzed three cohorts of healthy adults across different continents, describing relatively low microbial diversity in males and elderly people [20]. 

Thus, although microbiome disruptions are common in the ICU and general patterns are observed, the range of factors contributing to these alterations in ICU patients results in highly individual patterns of intestinal microbiota [9]. 

### 2.2. Potential Negative Consequences of Gut Microbiota Disruptions

Dysbiosis of the gut microbiome, and specifically overgrowth by pathobionts (commensal microbes with pathogenic potential), has been associated with adverse clinical outcomes. For example, in eight patients that underwent allogeneic hematopoietic cell transplant, intestinal domination by Proteobacteria or Candida resulted in translocation and subsequent invasive bacterial and fungal infections [21]. In a larger study that followed 708 recipients of allogeneic hematopoietic cell transplant, it was found that overrepresentation of Gram-negative bacteria was strongly associated with the development of bloodstream infections [22]. A study in 301 critically ill patients found that Enteroccocus domination (>30% relative abundance) of the gut microbiome was associated with a 19% increased probability of death, significant after correction for disease severity [23]. 

In addition, gut microbiome perturbations potentially have negative long-term health consequences and could be of clinical relevance following a hospitalization and ICU stay. Large observational studies described associations between presumed disrupted microbiota (based on antibiotic exposure or diseases associated with dysbiosis) and subsequent increased risks of sepsis [24,25]. One observational study from the US used data from over 12 million hospitalized patients and found a doubled risk of severe sepsis in the 90 days following hospitalization in patients exposed to ≥4 antibiotic classes or ≥14 days of antibiotic therapy, compared to those without antibiotic exposure [24]. Exposure to high-risk antibiotics (e.g., third- or fourth-generation cephalosporin or fluoroquinolones) was associated with a 1.65-fold greater risk of severe sepsis in the 90 days following discharge [24]. Although the aforementioned studies suggest a link between intestinal microbiota disruptions and critical illness in humans, whether this links implies a causal relation and—most importantly—a modifiable one remains undetermined. 

### 2.3. Mechanisms Underlying the Beneficial Role of Probiotics in Critical Illness

Probiotics are hypothesized to reconstitute the disrupted intestinal microbiome and may provide health benefits through two main mechanisms. First, probiotics would inhibit pathogen growth or replace pathogenic bacteria with non-pathogenic bacteria (the probiotic) and create a more favorable microbial environment in the stomach and gut. Thus, oropharyngeal colonization by pathogenic bacteria could be prevented, thereby diminishing the risk of pneumonia caused by micro-aspiration. Moreover, transloca-tion of intestinal bacteria to the blood and distant organs might be avoided by replac-ing pathogenic gut bacteria [26]. Second, a re-established microbiome could provide health benefits by influencing immune responses outside the gut [13,27]. 

However, as the mechanisms underlying the role of gut microbiota perturbation in critical illness are not yet fully understood, the exact mechanisms of action of most probiotics are not yet known either. Animal models revealed some potential mechanisms through which gut microbiota disruptions result in reduced colonization resistance against pathogens and immune derangements. As an example, in health, commensal bacteria prevent the expansion of pathogens through a competition for nutrients, enhancement of immunoglobulin A production, and by stimulating the release of antimicrobial peptides such as regenerating islet-derived protein IIIγ (REGIIIγ) from epithelial cells [28,29]. In addition, commensal-derived short-chain fatty acids (SCFAs) serve as the main nutrient of gut enterocytes, which maintain intestinal barrier function, thereby protecting against systemic dissemination of pathogenic bacteria. During critical illness, the decrease of commensal bacteria leads to a loss of colonization resistance and increased gut permeability, resulting in an overgrowth of harmful microbes and subsequent translocation to blood and distant organs, specifically the lungs and brain [30,31]. Thus, probiotic supplementation might re-establish the disrupted intestinal microbiome and provide colonization resistance against pathogens.

The beneficial effects of a reconstituted microbiome extend beyond the intestine, through the production of immunomodulatory metabolites. Gut derived SCFAs can, for instance, affect the immunological environment in the lung and increase the bactericidal activity of alveolar macrophages [32]. Another microbial metabolite, D-lactate, translocates from the gut to the liver through the portal vein and promotes pathogen clearance by Kupffer cells (the resident macrophages of the liver) [33]. In addition, murine studies suggested the involvement of gut microbiota in complications of critical illness such as acute kidney injury induced by ischemia-reperfusion, acute respiratory distress syndrome (ARDS) and liver injury [30,34,35]. Together, probiotics are hypothesized to prevent the detrimental consequences of gut dysbiosis and support a healthy enteric and systemic immune response [13]. 

However, whether commonly used probiotics actually approximate these functions of commensal microbiota, and whether these mechanisms are of equal importance in humans—where the microbiome is much more complex, and circumstances are not standardized—remains somewhat speculative. In a randomized controlled trial aiming to translate such preclinical evidence to healthy humans, gut microbiota disruption with broad-spectrum antibiotics had no effect on the surrogate markers of sepsis (e.g., vital signs and systemic cytokine responses) upon intravenous lipopolysaccharide injection [36]. Similarly, existing interindividual differences in gut microbiota composition were not associated with variation in cytokine responses (TNF-α, IL-6, IL-8 and IL-10) during the same model of experimental endotoxemia [37]. This underscores the notion that the gut microbiota is just one of the many factors that regulate the systemic immune response, and also highlights the difficulty of translating findings from animals to humans. 

## 3. Microbiome Modulation in the ICU

### 3.1. Preclinical Data on the Efficacy of Probiotics

Preclinical findings, specifically mouse models of severe infection, have further built the rationale for probiotic approaches in the ICU. For example, administration of *Lactobacillus* and *Bifidobacterium* blunted the pro-inflammatory response, decreased lung injury and improved survival in a mouse model of sepsis induced by cecal ligation and puncture [38,39]. In comparable sepsis models, mice pretreated with *L. rhamnosus GG* showed improved survival compared to controls [40]. In more comprehensive follow-up studies, the same research group showed that pretreatment with *L. rhamnosus GG* limited sepsis-induced dysbiosis, improved read-outs of the intestinal barrier function, decreased inflammatory cytokine levels, and prevented changes in some fecal metabolites, such as lysophosphatidylcholine and eicosatetraenoic acid lipids of which the (patho)physiological relevance remain uncertain [41,42]. In neonatal mice, administration of *L. murinus* protected against gut overgrowth of the pathobiont *Klebsiella pneumoniae*, thereby preventing subsequent systemic translocation and late-onset sepsis. Interestingly, only selected lactobacilli, namely, *L. murinus,* were effective probiotics, while the commonly used commercial strains, *L. rhamnosus GG* and *L. plantarum,* did not protect against dysbiosis [43]. Together, experimental data in murine models of sepsis showed beneficial effects of (specific strains of) probiotic intervention in modulating the gut microbiome, although the exact mechanisms largely remain to be elucidated. 

### 3.2. Prevention of Ventilator-Associated Pneumonia

The negative outcomes associated with dysbiosis in the ICU, together with the beneficial effects of probiotics in murine studies, have provided the rationale for probiotic intervention to prevent secondary infections in the critically ill. Specifically, in recent years most attention has be paid to the use of probiotics for the prevention of VAP.

VAP is defined by the American Thoracic Society as hospital-acquired pneumonia in patients that have been on mechanical ventilation for at least 48 h [44]. VAP is reported to affect 10–25% of all mechanically ventilated patients, with the incidence ranging from 2 to 15 cases per 1000 ventilator-days [45]. The pathogenesis of VAP is complex and multi-facetted, involving an interplay between (endogenous) bacteria, the detrimental physiological effects of intubation, and decreased immunological resilience during critical illness [46]. The endotracheal tube facilitates the entry of pathogenic bacteria—either translocated from the digestive tract or via inhalation—to the lower respiratory tract through micro-aspiration, biofilm formation and impaired mucociliary clearance. A dysregulated immune response during critical illness and mechanical ventilation further contributes to the development of VAP, including a role being played by the decreased phagocytic activity of macrophages [47], impaired type I interferon signaling [48], and neutrophil dysfunction [49]. Overall, the translocation of bacteria from the digestive tract to the lungs might be a core mechanism in VAP [50], and altering the composition of the gut microbiome through probiotics aims at combatting this mechanism.

Over the last decades, a multitude of trials have been performed in this rapidly expanding field. A recent meta-analysis pooled the results of nine randomized controlled trials, together reporting on 1127 patients (564 receiving probiotics and 563 receiving placebo), all investigating probiotic intervention in the ICU, with the primary aim of reducing the incidence of VAP [51]. The studies included used myriad probiotics, including *Lactobacillus*, *Bifidobacterium* and *Streptococcus* spp., and two specific probiotic formulas (containing *Bacillus* and *Enterococcus* spp., or *Pediococcus* and *Lactobacillus* spp.).

An overall positive effect of probiotic intervention was found with a lower incidence of VAP (odds ratio 0.70, confidence interval 0.56–0.88), shorter duration of mechanical ventilation (mean difference of 3.75 days), shorter ICU stay (mean difference of 4.20 days) and lower in-hospital mortality (odds ratio 0.73, confidence interval 0.54–0.98). The total length of hospital stay was unaffected. This systematic review assessed several forms of bias and performed subgroup analyses, which did not reveal apparent publication bias, nor significant differences between trials with a high vs. low risk of bias, or between trials undertaken in a trauma vs. mixed population of patients. The studies included in the meta-analysis did show heterogeneity in terms of the definition of VAP and in the intervention, as some studies employed a single-strain probiotic (such as *L. rhamnosus*), while others used multiple probiotics (e.g., a combination of three *Lactobacillus* species and *B. bifidum*), or a synbiotic product (e.g., ‘Synbiotic 2000Forte’ which contains *Pediococcus* and *Lactobacillus* spp. along with inulin, betaglucan, pectin, and resistant starch as the prebiotic). Notably, the route, timing, and length of intervention was also variable. The conclusion of this meta-analysis—that VAP incidence was lower in the probiotic group—is in line with several earlier systematic reviews [52,53,54,55,56]. Together, almost all systematic reviews conclude that any result must be interpreted with caution. The heterogeneity in cohort characteristics, type of probiotic intervention and study design warranted a large, multi-center randomized controlled trial [51,52,53,54,55,56]. Recently, such a trial has been published.

In the randomized, placebo-controlled PROSPECT trial in 44 hospitals across three countries, Johnstone et al. investigated whether probiotic administration could lower the incidence of VAP [57]. The study included 2653 patients in the ICU—expected to be on mechanical ventilation for at least 72 h—split evenly between 1 × 10^10^ colony forming units of *L. rhamnosus GG* or placebo twice daily, for a period of sixty days or until discharge. The results were clear: the probiotic intervention did not lower the incidence of VAP (21.9% in the probiotic group, 21.3% in the placebo group). Furthermore, no differences were found when they used alternative definitions for pneumonia. The discrepancy between these findings and results from previous studies and meta-analyses, often including *L. rhamnosus* as a probiotic intervention too, is remarkable. This may be a product of the inter-study heterogeneity in terms of design and patient population, or differences between the probiotic formulae. The importance of this heterogeneity is highlighted by a recent, smaller study with a different design and in this placebo-controlled trial, 112 multi-trauma patients—expected to be on mechanical ventilation for at least 10 days—were randomized between either a probiotic formula (consisting of *L. acidophilus*, *L. plantarum*, *B. lactis* and *Saccharomyces boulardii*) or placebo twice daily for two days [58]. The incidence of VAP (11.9% vs. 28.3%, respectively) and sepsis (6.8% vs. 24.5%, respectively) was significantly lower in the probiotic group, while the length of hospital and ICU stay were also reduced. Notably, the study stopped prematurely and included less than half of the intended number of patients. Although this limitation may preclude robust conclusions, the contrast between the findings of these studies is stark and may in part be explained by a different patient population and the use of a multi-strain probiotic formula. Overall, the current level of evidence tempers the initial enthusiasm on the use of probiotic therapy for the prevention of VAP, and more work is needed to identify which probiotic intervention may be beneficial for specific patient groups.

### 3.3. Other Indications in the ICU

While the prevention of VAP has been the main focus in probiotic research, several other outcome measures have also been investigated including diarrhea, other infections, length of hospital stay and mortality. A recent placebo-controlled randomized controlled trial in 218 Australian ICU patients by Litton et al. assessed the effect of early daily *Lactobacillus plantarum* 299v supplementation [59]. The primary outcome was days alive and out of hospital to day 60, a composite endpoint of death, hospital length of stay and hospital re-admissions. Early and sustained administration of *L. plantarum* 299v did not improve the primary outcome measure (49.5 (IQR 37–53) in the probiotic group and 49 (IQR 43.8–53) in the placebo group, *p* = 0.55) [59]. Several subgroup analyses, including the evaluation of antibiotic treatment, the presence of sepsis and type of ICU admission, did not reveal significant differences either. This is in line with the recent findings by Johnstone et al. that found no differences in ICU and hospital length of stay, or mortality [57]. Moreover, while a meta-analysis of 14 trials reporting on a total of 1233 critically ill patients found a reduction in infections following probiotic treatment (risk ratio 0.80, confidence interval 0.68–0.95) [55], the incidence of infections was not different between groups in the two recent trials (by Johnstone et al. and Litton et al.) [57,59]. The incidence of any infection was 31.4% in both the placebo and the probiotic group (hazard ratio 0.97, confidence interval 0.84–1.11) [57], and nosocomial infections occurred in 7.3% and 4.6 of the probiotic and placebo group patients, respectively (odds ratio 1.62, confidence interval 0.51–5.10) [59]. Together, as we noted for VAP, the results of recent high-quality trials appear to deviate from the conclusions of meta-analyses. 

Given the often detrimental effects of antibiotics on the gut microbiome and their wide use in ICU patients, multiple trials have investigated whether probiotics could mitigate the negative consequences of antibiotic perturbation such as antibiotic-associated diarrhea and *Clostridium difficile* infection. A meta-analysis of nine trials and 1259 ICU patients did not demonstrate a treatment benefit of probiotics on diarrhea (risk ratio 0.97, confidence interval 0.82–1.15) [55]. Likewise, in the aforementioned trial by Johnstone et al. there were no differences in the incidence of antibiotic-associated diarrhea (hazard ratio 1.02, confidence interval 0.93–1.15) or *C. difficile* infection (odds ratio 1.15, confidence interval 0.69–1.93) [57]; however, in meta-analyses including both out- and in-patients, rather than focusing solely on ICU patients, probiotics reduced the risk of *C. difficile* infection and antibiotic-associated diarrhea [60,61]. Among 13 trials enrolling 2454 participants with a high baseline risk of *C. difficile* associated disease (>5%), probiotics reduced the risk of *C. difficile* associated disease by 70%, but no significant effect of probiotics was found in trials with a lower baseline risk (≤5%) [60]. Due to the lack of conclusive high-quality evidence, probiotics are currently not included in treatment guidelines for *C. difficile* infections [62,63].

Since the start of the COVID-19 pandemic, multiple randomized-controlled trials assessing the potential role of probiotic treatment in COVID-19 have been registered [64]. Of those, only one investigates the effect of probiotics (*Streptococcus salivarius* K12 combined with *L. brevis*) in ICU patients with COVID-19 (clinicaltrials.gov: NCT05175833). Thus far, no results of this trial are available and the role of probiotics in critically ill COVID-19 patients remains unclear. 

Overall, current evidence does not unambiguously support the use of probiotics for the prevention or treatment of antibiotic-associated diarrhea and *C. difficile* infection in ICU patients. The identification of subgroups that could potentially benefit from probiotics is an important future challenge.

## 4. Current Challenges

### 4.1. Safety

In addition to the unclear efficacy, the implementation of probiotic treatment in the ICU has been hampered by safety concerns. These concerns stem in part from the frequently debated and re-analyzed results of the PROPATRIA study [65,66,67], a double-blind, placebo-controlled trial in which patients with predicted severe acute pancreatitis received either enteral probiotics (a combination of *three Lactobacillus* spp., two *Bifidobacterium* spp. and one *Lactococcus* spp.) or placebo. The probiotic treatment resulted in higher mortality (16%, 24 of 152 patients) compared to the placebo (6%, 9 of 144 patients), which was presumably—albeit still a subject of debate—caused by intestinal ischemia and translocation of gut bacteria to the bloodstream, resulting in multiorgan failure.

Although probiotic supplementation has earlier been associated with higher risks of sepsis and fungemia in critically ill patients [68], it was only recently shown that probiotics supplementation in pediatric ICU patients could result in the systemic translocation of probiotic bacteria. Epidemiological data of 22,174 ICU patients showed that patients receiving *Lactobacillus rhamnosus GG* were at increased risk of Lactobacillus bacteremia (6 out of 522 patients, compared to 0 out of 21,652). Whole-genome-based phylogeny analysis confirmed that *Lactobacilli* isolated from the blood of patients treated with probiotics were phylogenetically inseparable from the probiotic product [69]. Similarly, in the aforementioned trial by Johnstone et al. that investigated 2653 ICU patients, the incidence of adverse events (including the sequencing-confirmed presence of *Lactobacillus rhamnosus GG* in previously sterile sites) was significantly higher in the probiotic group (1.1% versus 0.1% in the placebo group) [57].

Together, these studies raise valid questions regarding the potential harm of probiotic supplementation in the critically ill, and a thorough examination of adverse effects is warranted. Of note, it was recently reported that out of 53 studies investigating probiotic, prebiotic or synbiotic intervention in hospitalized and/or critically ill patients, only 7 reported the number of serious adverse events per group [70].

### 4.2. Other Pitfalls in the Field

Despite the many links between microbiome disruption and adverse outcomes in the ICU, and the apparent beneficial effect of probiotics on mortality and inflammation in numerous animal models of severe infection, probiotic treatment has not unequivocally proven to be of clear clinical benefit in critically ill patients. Therefore, what challenges need to be addressed, in order for probiotics to reach their full clinical potential in the ICU (Table 1)?

First, practical issues such as dosage, treatment duration, timing and the effects of concurrent administration with antibiotics—potentially directly eliminating the administered bacteria—need to be considered and ideally standardized to improve the interpretation and comparability of RCTs. Next, microorganisms that are used as a probiotic should be adequately characterized in terms of their genome and functional repertoire, as strain level differences influence their health-promoting functions [43]. A recent study revealed enormous genetic and functional inter- and intra-species diversity within a single commensal gut family. Through whole-genome sequencing and gene annotation in 20 human donors, the authors found remarkable differences within the Lachnospiraceae family, which are likely to influence butyrate production of a specific strain and thereby its contribution to colonization resistance and the host’s mucosal immune response [71]. These findings indicate that proper genomic and metabolic analyses of microbes is essential to identify the strain-specific qualities that could be harnessed in effective new probiotics.

Furthermore, although an altered microbiome could be assumed to be a prerequisite for any beneficial effects of probiotics, the actual effect of probiotic supplementation on gut communities is very often not reported in human trials [72]. A systematic review found no effect of probiotics on the fecal microbiota composition of healthy adults in six out of seven randomized controlled trials [73]. Recently, two key studies described the effect of probiotics on the gut microbiome in much more detail. Zmora et al. described the impact of probiotics on the human gut mucosa-associated microbiome [74]. By characterizing the microbiome in mucosal stool samples before and during the administration of a placebo or an 11-strain probiotic preparation (existing of *Lactobacillus*, *Bifidobacterium*, *Lactococcus* and *Streptococcus* spp.), they found a transient and highly individualized effect of probiotics on the mucosal communities and the gut transcriptome—approximately half of the participants showed significantly higher abundances of probiotics in their gut mucosa, while others were not colonized by probiotics. This person-specific susceptibility to gut colonization by probiotics was associated with baseline host transcriptional and microbiome characteristics and could explain the high interpersonal variability in probiotic effects. Of significance, Suez et al. showed that a four-week administration of the same multi-strain probiotic formula after broad-spectrum antibiotic exposure resulted in a delayed microbiome reconstitution when compared to watchful waiting and autologous FMT [75]. Intestinal, mucosal and stool samples indicated that the probiotics inhibited the repopulation of the indigenous communities, both in terms of microbial diversity and transcriptional profile. These findings shed light on the longitudinal effects of probiotic intervention and indicate that temporarily boosting the gut microbiome with probiotics may result in a stunted recovery of the microbiome in the long-term. This previously underestimated trade-off is seldom taken into account in current studies and warrants an extended monitoring of the microbiome and outcome of patients treated with probiotics.

Finally, what constitutes a “healthy microbiome”—or similarly, dysbiosis [76]—remains ill-defined [77]. While a core human microbiome may exist, it is known that each individual carries a personalized microbial signature that evolves throughout life. The heterogeneous consequences of ICU treatment on gut microbiota composition and the person-specific gut mucosal colonization resistance against probiotics [8,70], highlight the need for personalized approaches to reconstitute the disrupted microbiome rather than a standardized, single-strain probiotic intervention in the highly diverse ICU population. In other words, one size will probably not fit all.

## 5. Future Perspectives

**Figure 1:**
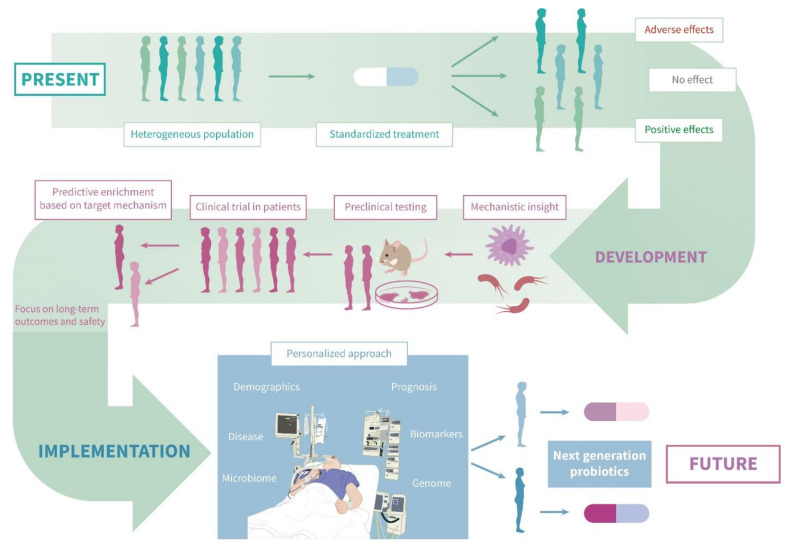
Current and future role of probiotics in the ICU. Current practice involves a standardized intervention in the highly diverse ICU population, with inconsistent clinical effects. A focus on a mechanistic understanding, combined with rigorous preclinical testing—including in healthy volunteers—can lay the groundwork for new probiotics with well-documented biological effects. The clinical efficacy of these next-generation probiotics should be tested in clinical trials with a focus on long-term outcomes and safety. Herein, dividing patients into specific subgroups (predictive enrichment) based on the target mechanism can increase the chance of finding positive effects. Eventually, the use of patient-specific data may allow clinicians to tailor probiotic treatment in the ICU to individual patients.

It is notable that although indirect evidence for the importance of the gut microbiome is abundant (associations with clinical outcome, in vitro work and mouse models), proven mechanistic links between gut microbiome changes and the (patho)physiology of critically ill humans remain absent. Nevertheless, many randomized-controlled trials have been performed over the last decade. The fact that probiotics are classified as food supplements, and not as medication, could perhaps partly explain this early transition to human intervention trials. A focus on the mechanistic, causal effects of specific features of the human microbiome is advised to be the basis for future interventional trials (Figure 1) [78].

In recent years, several preclinical studies have described novel live microorganisms that have not been used to promote health to date. These non-standard probiotics—also known as next-generation probiotics [79]—often comprise gut commensals rather than the currently used *Lactobacillus* or *Bifidobacterium* species and might affect the gut microbiome and protect against infections. For example, murine studies demonstrated that a combination of four gut commensals (*Bacteroides sartorii*, *Parabacteroides distasonis*, *Clostridium boltea* and *Blautia producta*) restored colonization resistance against vancomycin-resistant Enterococci through cooperativity between these commensals [80]. In addition, *Clostridium scindens* (another gut commensal) could reduce enteric colonization by *C. difficile* through synthesizing *C. difficile*-inhibiting metabolites from bile salts [81]. 

Studies could also focus more on the prevention of microbiota disruption by antibiotics, aside from reconstituting the microbiome after iatrogenic dysbiosis. In this context, a recent investigation screened potential antidotes that may specifically mitigate the collateral damage of antibiotics on commensals [82]. By analyzing a library of 1197 pharmaceuticals, it was reported that an anticoagulant drug (dicumarol), an uricosuric agent (benzbromarone) and two non-steroidal anti-inflammatory drugs (tolfenamic acid and diflunisal) could protect *Bacteroides* species from the negative effects of erythromycin and doxycycline. Importantly, it was shown in human-stool-derived communities and gnotobiotic mice (i.e. animals containing only known microorganisms) that these antidotes did not affect antibiotic efficacy against the pathogens for which erythromycin and doxycycline were prescribed [82]. Further development of these next-generation probiotics and antidotes could result in new therapeutics that limit antibiotic-induced damage to the microbiome, enhance colonization resistance and reduce (antibiotic-resistant) infections [28]. Ideally, future trials assessing such interventions should comprehensively measure the effects on the microbiota composition over an extended period of time.

## 6. Conclusions

Altogether, we can conclude that the field of microbiota research has comprehensively shown that the gut microbiome is severely disrupted in critically ill patients in the ICU. The resulting dysbiosis has been associated with worse clinical outcomes, re-infections and re-admissions, but causal relationships remain elusive. Similarly, there are strong indications from experimental data that probiotic intervention may improve outcomes in models of severe infection, but the underlying mechanisms are still unclear. Substantial heterogeneity between randomized controlled trials, concerns about safety and a recent high-quality trial with negative results with regards to VAP prevention reflect that a beneficial role for probiotics in the ICU remains uncertain. Future experimental and clinical studies focused on mechanistic evidence, are needed to determine how the full potential of the microbiome in terms of its diagnostic and therapeutic value can be unlocked in the ICU setting. While we may have to go back to the drawing board and rethink our approach, microbiome modulation in intensive care remains a promising clinical tool to improve long-term outcomes.

## Figures and Tables

**Table 1:** Current challenges for probiotics in the ICU.

**Efficacy**	While the majority of meta-analyses find a positive effect, the negative results of the recent PROSPECT trial cast doubt on the efficacy of probiotics for preventing ventilator-associated pneumonia [57].
**Safety**	Overall lack of safety reporting, coupled with recent reports of probiotic bacteremia, together warrant increased attention for monitoring potential harm.
**Mechanisms**	Causal links between probiotic intervention and improved outcome in experimental models remain largely elusive.
**Microbiome Effects**	Microbiome diversity and composition are often not among the (secondary) outcome measurements in clinical trials, which cloud our understanding of the (long-term) effects of probiotics on gut microbiota.
**Heterogeneity**	Gut microbiota, and the negative effect of antibiotics thereon, show inter-individual differences which may call for more personalized therapy.
